# Improved Local Anesthesia

**Published:** 1898-04

**Authors:** 


### Improved Local Anesthesia.

Operations on fingers and toes, ingrowing nails, panaritium, etc., are the most difficult to anesthetize with the Schleich method, and yet they are the most frequent in general practice. Oberst's method is simple and effective for these cases. The finger or toe is first raised to expel the blood, and the central end, as close to the field of operation as possible, is wound once or twice with an elastic cord, the ends not tied but held with a clamp. Cocain is then injected in the four sides of the finger, directed toward the tip, just below the ligature. The anesthesia is complete in three to ten minutes, tested with a prick, and lasts as long as the circulation in the part is arrested. This method has been followed at Breslau in 124 cases of the kind with such satisfactory results, that Honigmann recommends it warmly in the *Cbl. f. Chir.*

